# Cholesterol Ester Storage Disease in Two Field Spaniels With Lysosomal Acid Lipase Deficiency

**DOI:** 10.1111/jvim.70223

**Published:** 2025-08-26

**Authors:** Pernilla Syrjä, Mathilda M. H. Ylenius, Matilda Kråkström, Alex M. Dickens, Elina Rautala, Anna Huupponen, Anders Eriksson, Sanna J. Viitanen

**Affiliations:** ^1^ Department of Veterinary Biosciences, Section for Veterinary Pathology and Parasitology, Faculty of Veterinary Medicine University of Helsinki Helsinki Finland; ^2^ Department of Equine and Small Animal Medicine, Faculty of Veterinary Medicine University of Helsinki Helsinki Finland; ^3^ Turku Bioscience Centre University of Turku Turku Finland; ^4^ Department of Chemistry University of Turku Turku Finland; ^5^ Carditech Espoo Finland

**Keywords:** canine, dog, lipid metabolism, lipidomic

## Abstract

Cholesterol ester storage disease (CESD) is a rare genetic lysosomal storage disorder resulting from lower lysosomal acid lipase (LAL) activity. LAL is an essential enzyme required in intracellular lipid metabolism, and deficiency results in disability to properly break down and utilize lipids and in the accumulation of especially cholesterol esters in many organs such as the liver, spleen, and bone marrow. This case report describes clinical findings, LAL activity measurement, blood and liver tissue lipidomic changes, as well as pathological findings in two unrelated Field Spaniels with LAL deficiency and CESD.

**Abbreviations**
AFOS: alkaline phosphatase
ALAT: alanine aminotranferase
ASAT: aspartate aminotransferase
BCS: body condition score
CE: cholesterol ester
CESD: cholesterol ester storage disease
LAL: lysosomal acid lipase
LRR: laboratory reference range

## 1 Case Description

### 1.1 Case 1

**FIGURE 1:**
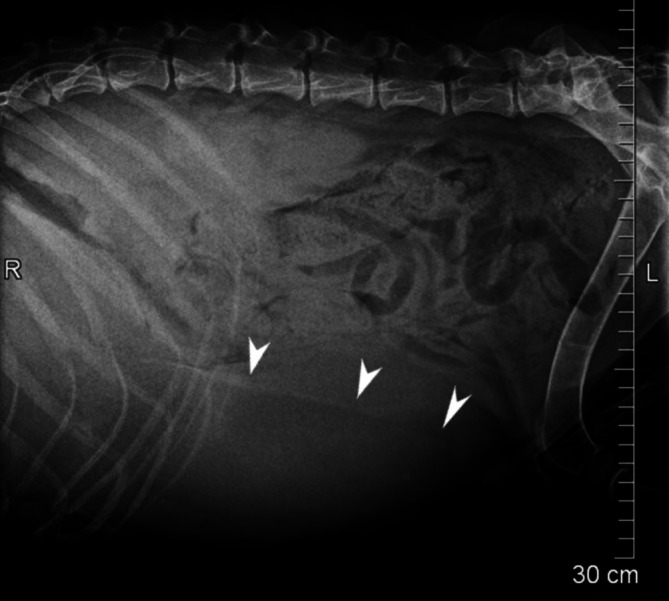
Laterolateral radiograph from a 2.7 years old male field spaniel with cholesterol ester storage disease and marked hepatomegaly (liver silhouette marked with arrows).

**FIGURE 2:**
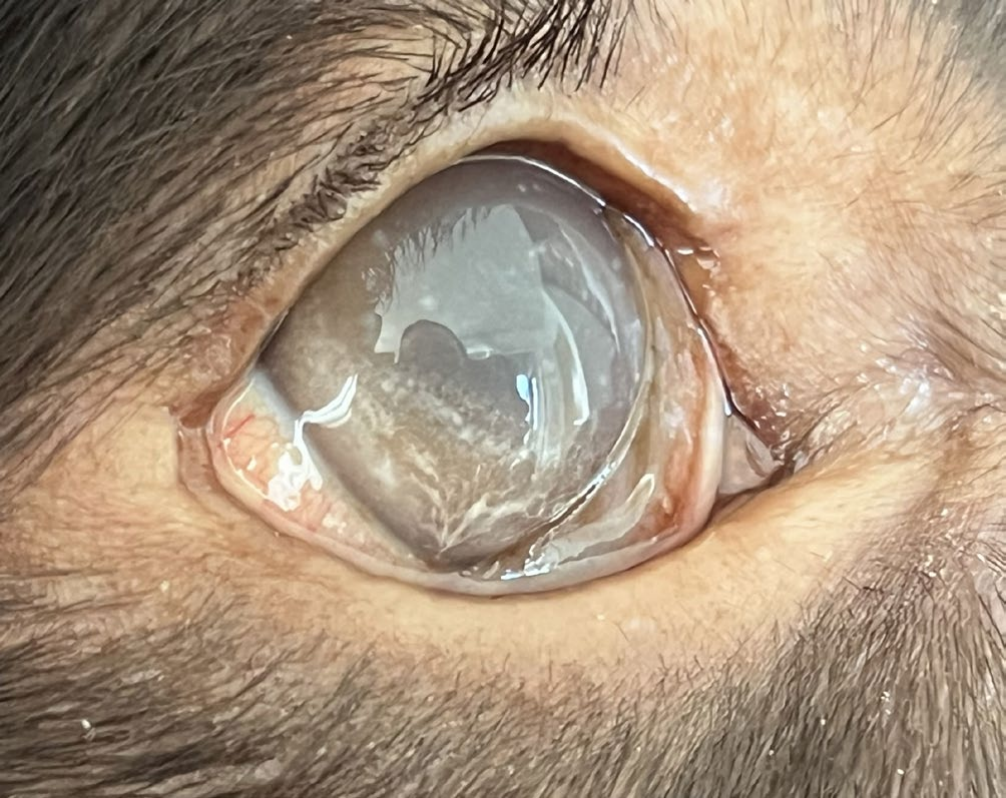
Photograph of the eye of a 3.7 years old male field spaniel with cholesterol ester storage disease and corneal lipid accumulation.

A 2.8‐year‐old, intact male, Field Spaniel was referred to the University Veterinary Teaching Hospital in Helsinki due to weight loss and hepatomegaly (Figure 1). The dog was initially examined at the referring veterinarian at 6 months of age because of corneal lipid accumulation (Figure 2) and episclerokeratitis, and approximately one year later, weight loss and a distended abdomen had been noted. On clinical examination, the dog was thin (body condition score [BCS] 2/9) and there was mild muscle loss. The abdomen was distended, and cranial abdominal organomegaly was suspected based on palpatory findings. Serum biochemistry analysis (Konelab 30i, Thermo Scientific, Vantaa, Finland) revealed moderate hypoalbuminemia (23.2 g/L, laboratory reference range [LRR] 30–41 g/L), moderate hypocholesterolemia (2.0 mmol/L, LRR 3.7–9.8 mmol/L) and mild hypertriglyceridemia (1.25 mmol/L, LRR 0.29–1.17 mmol/L). Serum liver enzymes (alanine aminotransferase [ALAT] and alkaline phosphatase [AFOS]) and bilirubin, as well as coagulation variables (pT and aPPT, Sysmex CS‐5100, Sysmex Corporation, Kobe, Hyoko, Japan) and blood ammonium (Blood Ammonia Checker II, Menarini Inc. Florence, Italy) were within LRR.

Abdominal ultrasonographic examination (EPIQ 7G, Koninklijke Philips N.V., Eindhoven, Netherlands) showed marked hepatomegaly with rounded liver edges, increased echogenicity, and heterogenic parenchyma with multiple small hyperechoic foci. In the spleen, multiple hypoechoic and heterogenic lesions were noted. Additionally, a single markedly enlarged abdominal lymph node with normal echogenicity was detected.

**FIGURE 3:**
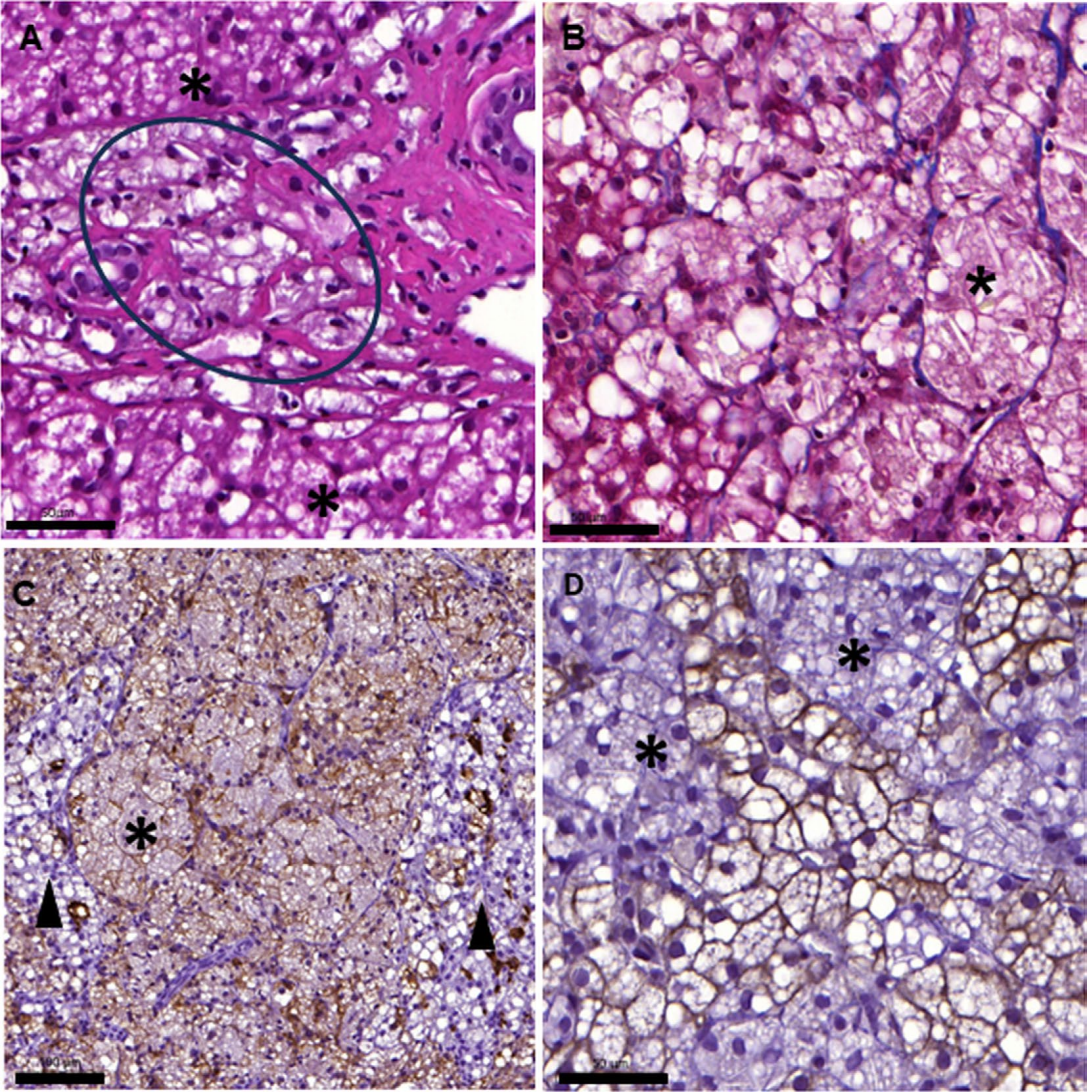
(A–D) Histopathology images from liver biopsy samples obtained from a 3.0 years old male field spaniel (*Case 1*) and a 1.7 years old female Field Spaniel (*Case 2*) with cholesterol ester storage disease. (A) The liver shows moderate lipidosis of hepatocytes (*), and interstitial as well as lobular nests of lipid‐containing macrophages (circle). *Case 2*, hematoxylin and eosin stain. (B) Infiltration of fat‐ and cholesterol‐filled macrophages (*), supported by fine fibrillar collagen (blue). A small area of remnant liver parenchyma, showing lipidosis is present in the lower left corner. *Case 1*, Masson trichrome stain. (C) Overview of the extensive macrophage infiltration. Nodular, expansive foci of Iba‐1 positive, macrophages with abundant foamy cytoplasm (*) are visible between separated hepatic cords (arrowhead), where single Kupfer cells stain positive. *Case 1*. Iba‐1 immunohistochemistry (IHC). (D) The cellular lesion pattern. Hepatocytes remain as cytokeratin‐positive strands, in between the macrophage nests (*). *Case 2*. Cytokeratin IHC. Size bar for A, B and D 50 μm and for C 100 μm.

Liver biopsies were obtained laparoscopically, and histopathological findings included severe hepatic panlobular lipidosis, evident as smooth, round clear vacuolization in the hepatocellular cytoplasm (Figure 3A). Multifocal to coalescing infiltrates of macrophages, containing fatty material and cholesterol clefts in their cytoplasm, were present in the hepatic interstitium and lobuli. Macrophages were packed into nests surrounded by mild fibrosis (Figure 3B). The macrophage infiltration occupied up to half of the biopsy area when visualized using immunohistochemical staining (Figure 3C,D).

The dog was treated with a low‐fat diet and hepatic supportive treatment (ursodeoxycholic acid and antioxidants). During the follow‐up period of one year, both weight loss and hepatomegaly gradually progressed, and serum albumin decreased (20.6 g/L, LRR 30–41 g/L). At the last control visit at the age of 3.7 years, a mild elevation of ALAT (83 U/L, LRR 18–77) and a moderate increase in ASAT (228 U/L, LRR 17–54) were noted, but bilirubin remained normal. The dog was euthanized at the age of 4.1 years due to lethargy, cachexia, progressive abdominal enlargement, and chronic diarrhea.

### 1.2 Case 2

A 1.7‐year‐old, intact female Field Spaniel was initially presented at the referring veterinarian at six months of age due to corneal opacification and one month later for diarrhea and weight loss. Serum biochemistry analysis (Catalyst One Chemistry Analyzer, IDEXX Laboratories Inc., Westbrook, Maine, US) revealed mild hypoalbuminemia (25 g/L, LRR 28–43 g/L), borderline low cholesterol (3.6 mmol/L, LRR 3.6–10.3 mmol/L), and elevated liver enzyme concentrations (ALAT 224 U/L, LRR 25–122 U/L; AFOS 652 U/L, LRR 14–147 U/L; aspartate aminotransferase [ASAT] 270 U/L, LRR 14–59). Marked hepatomegaly was noted in the abdominal ultrasound examination and liver biopsies were obtained via ultrasound‐guided percutaneous core biopsy. Histopathological findings included hepatic lipidosis comparable to that of *Case 1*, with moderate infiltration of fat‐ and cholesterol‐containing macrophages in the hepatic parenchyma and the interstitium.

The dog was treated with a low fat diet and hepatic supportive care (ursodeoxycholic acid). During the follow up, the dog developed chronic small bowel diarrhea and progressive weight loss and was euthanized at the age of 2.1 years due to cachexia, lethargy, and marked hepatosplenomegaly. At the time of euthanasia, there was moderate hypoalbuminemia (19.8 g/L, LRR 30–41 g/L), mild hypocholesterolemia (3.4 mmol/L, LRR 3,7–9,8 mmol/L), mild hypertriglyceridemia (1.33 mmol/L, LRR 0.29–1.17 mmol/L) and mild to moderate liver enzyme increases (AFOS 172 U/L, LRR < 95; ALAT 238 U/L, LRR 18–77; ASAT 162 U/L, LRR 17–54), yet normal bilirubin and ammonia.

### 1.3 Post Mortem Findings

**FIGURE 4:**
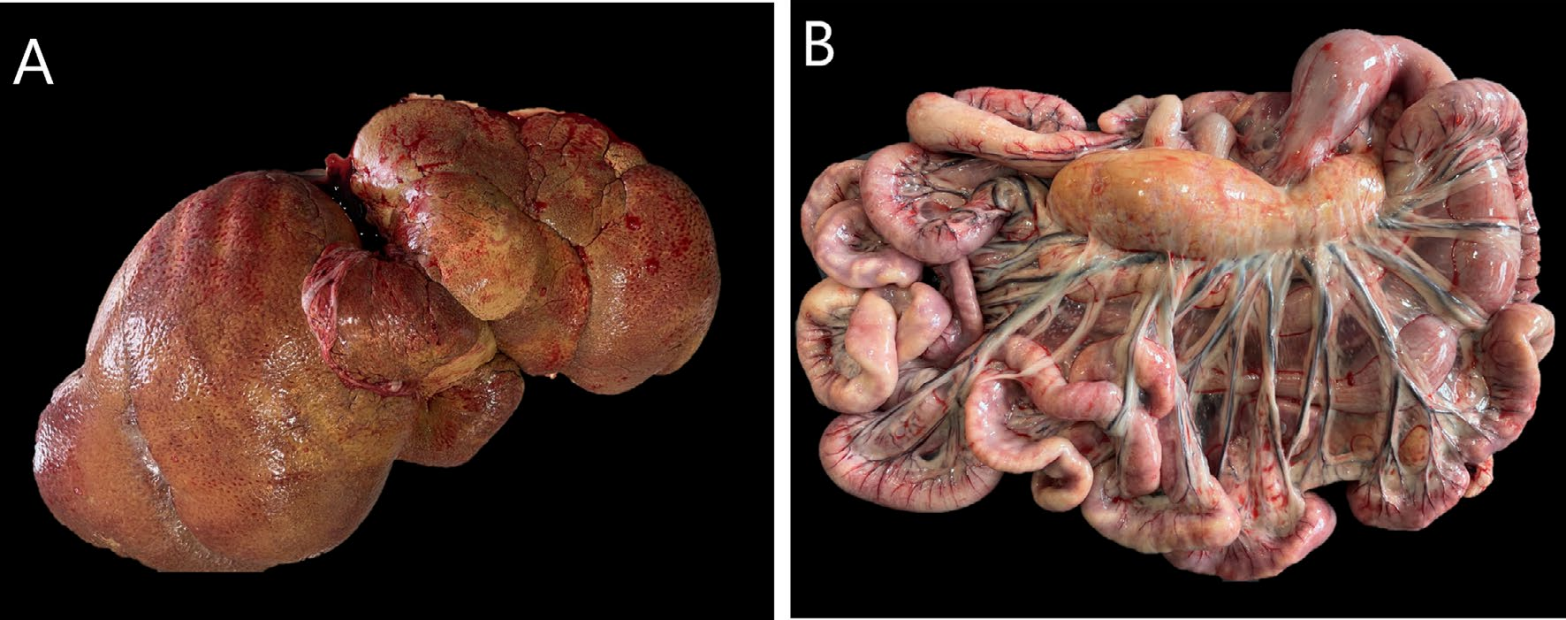
(A, B) Post‐mortem images from a 2.1 years old female Field Spaniel (*Case* 2) with cholesterol ester storage disease, showing marked hepatomegaly (A) and mesenteric lymphadenopathy (B).

At post mortem examination, both dogs showed extreme hepatomegaly (Figure 4A, liver weight 5.6 kg, 27% of body weight in *Case 1*, 3.6 kg, 24% of body weight, in *Case 2*), splenomegaly, keratoconjunctivitis with suspected limbal corneal lipid deposits, and severe generalized lymphadenopathy (Figure 4B). Histologically, cholesterol‐ and fat‐containing macrophages were present as multifocal to coalescing accumulations within the interstitium and parenchyma of the liver and spleen. Lymph node architecture was severely affected by numerous fatty macrophages. Similar macrophage accumulations were present subpleurally and peribronchially in the lungs, within the intestinal lamina propria, adrenal cortex, and bone marrow. Sparse, small infiltrations with macrophages containing fatty material and cholesterol were present within the myocardium, choroidal plexi, superficial cornea, and ocular ciliary body. Scattered single macrophages with similar cytoplasmic storage were present perivascularly in the dermis. A mild to moderate chronic lymphoplasmacytic inflammation accompanied the macrophages accumulating in the gastrointestinal mucosa, choroid plexi, heart, and cornea.

### 1.4 Lysosomal Acid Lipase Activity

Lysosomal acid lipase (LAL) activity was measured from venous EDTA‐blood using dried blood spot cards. A clinical mass spectrometry enzyme assay validated for humans was used (ARCHIMEDlife Laboratories, Vienna, Austria). LAL activity was measured from both affected dogs as well as from four privately owned healthy control dogs (median age 3.1 years, range 2.5–5.1 years, details in Data S1) with normal clinical examination as well as normal blood hematology and serum biochemistry analysis.

The LAL activity was markedly lower in both affected dogs (6.7 μmol/L/h in *Case 1* and 5.8 μmol/L/h in *Case 2*) when compared to healthy controls (median 143.2 μmol/L/h, range 94.4–261.2 μmol/L/h). LAL activity in both affected dogs was below the human threshold for the diagnosis of LAL deficiency (laboratory established cut‐off < 30 μmol/L/h).

### 1.5 Lipidomic Analysis

Two EDTA plasma samples from both affected dogs at two different time points 278 days (*Case 1*) and 150 days (*Case 2*) apart as well as single samples from four control dogs were stored at −80°C and shipped on dry ice to the University of Turku, Turku Metabolomics Centre for lipidomic analysis. *Dog 1* was fed a conventional diet at the first sampling and a low‐fat diet as well as silymarin at the second sampling. *Dog 2* was fed a low‐fat diet at both samplings. All control dogs were fed a conventional diet at the time of blood samplings, and all dogs were fasted before sampling. Additionally, liver biopsies from affected dogs (*Case 1* laparoscopic sampling at the time of diagnostic workup, conventional diet at sampling, no antioxidant medications; *Case 2* sampling immediately after euthanasia, low fat diet at sampling, no antioxidant medication) and four control dogs (sampling immediately after euthanasia, conventional diet at sampling) were stored at −80°C and shipped on dry ice for lipidomic analysis. Control liver biopsies were obtained from dogs (median age 11.6 years, range 0.8–15.0 years; median weight 14.3 kg, range 5.6–24 kg, details in Data S2) euthanized at the University of Helsinki Small Animal Teaching Hospital for reasons unrelated to liver disease and donated for teaching and research purposes.

**FIGURE 5:**
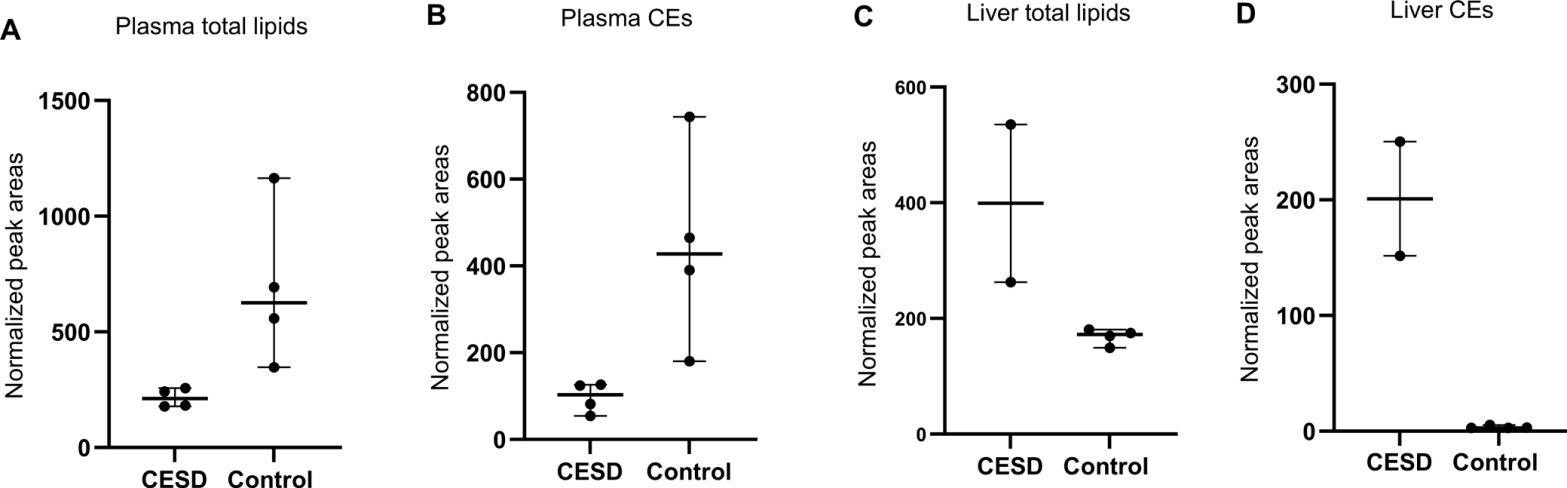
(A–D) Scatterplots showing the median and 95% confidence intervals as well as the individual values for total amount of (A) plasma lipids and (B) plasma cholesterol esters (CE) as well as total amount of liver tissue (C) lipids and (D) CEs in two Field Spaniels with cholesterol ester storage disease (plasma *n* = 4, two different time points for each dog; liver *n* = 2) and in healthy control dogs (*n* = 4, both plasma and liver tissue). The total amount of lipids and cholesterol esters (CEs) was calculated as the sum of normalized peak areas (NPAs) for all identified lipids and all identified CEs using mass spectrometry and compared between cases and controls (Mann–Whitney test). The total amount of plasma lipids and plasma CEs was lower in cases (median 211.9 NPAs and median 103.2 NPAs) compared to controls (median 625.7 NPAs and median 427.7 NPAs; *p* = 0.029 and *p* = 0.029, respectively), while total liver tissue lipids and CEs was higher in affected dogs (median 399.1 NPAs and median 201.0 NPAs) when compared to controls (median 172.4 and median 3.0 NPAs, respectively).

**FIGURE 6:**
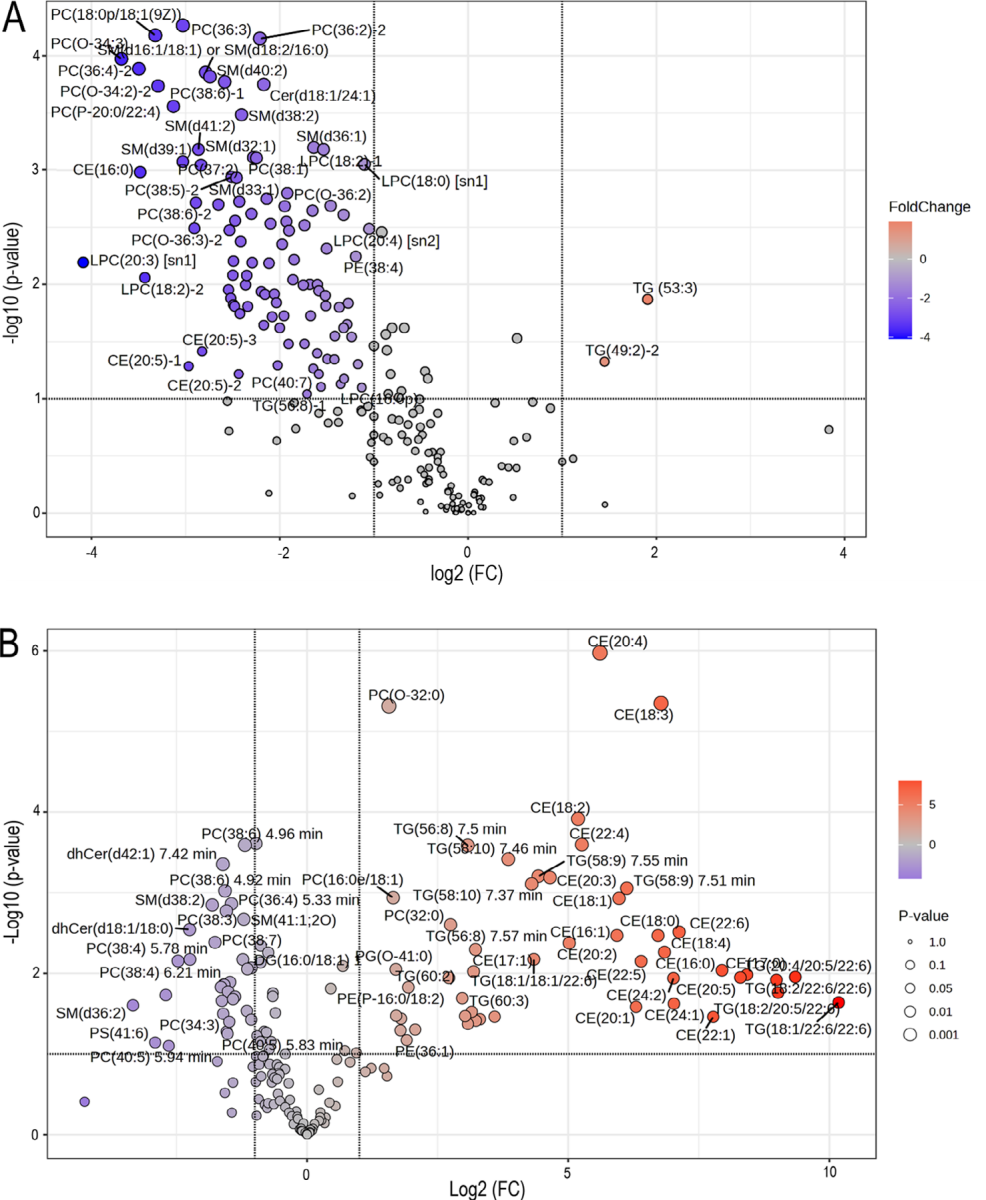
(A, B) Volcano plots demonstrating the type of plasma (A) and liver tissue (B) lipids, which changed most significantly between cases of cholesterol ester storage disease in two Field Spaniels (plasma *n* = 4, two samples at different time points for both dogs; liver biopsies, *n* = 2, additionally a calculated average of the two samples was used as a third measurement to enable statistical analysis) when compared to healthy control dogs (*n* = 4 both plasma and liver). (A) The plasma lipids that showed the most significant differences between the case and control groups included phosphatidylcholine (PC; 18:0p/18:1), PC (36:3), PC (36:2), PC (0–34:3), and sphingomyelin (SM; 34:2). Except for triacylglycerol (TG; 53:3) and TG (49:2), all significantly altered lipids were found to be lower in the case group compared to the controls. (B) In liver tissue, especially cholesterol esters (CE) and TGs were increased in cases when compared to controls and PC (O‐32:0), CE (20:4) and CE (18:3) were most significantly increased. The magnitude of the change (FC, fold change) is indicated in x‐axis and with colors. A positive FC indicates a higher lipid concentration in cases when compared to controls. The statistical significance of the change (*p*‐value) is indicated in y‐axis and with the size of the markers. In order to scale the data to fit on the graph the log values are displayed. The horizontal line represents a *p*‐value = 0.1 and the vertical lines represents a 2‐fold increase or decrease. CE, cholesteryl ester; Cer, ceramides; DG, diacylglycerol; dhCER, dihydroceramide; HexCer, hexosylceramides; Hex2Cer, dihexosylceramide; LPC, lysophosphatidylcholine; PC, phosphatidylcholine; PC‐O, ether‐linked phosphatidylcholine; PE, phosphatidylethanolamine; PE‐O, ether‐linked phosphatidylethanolamine; PI, phosphatidylinositol; SM, sphingomyelin; TG, triacylglycerols.

**FIGURE 7:**
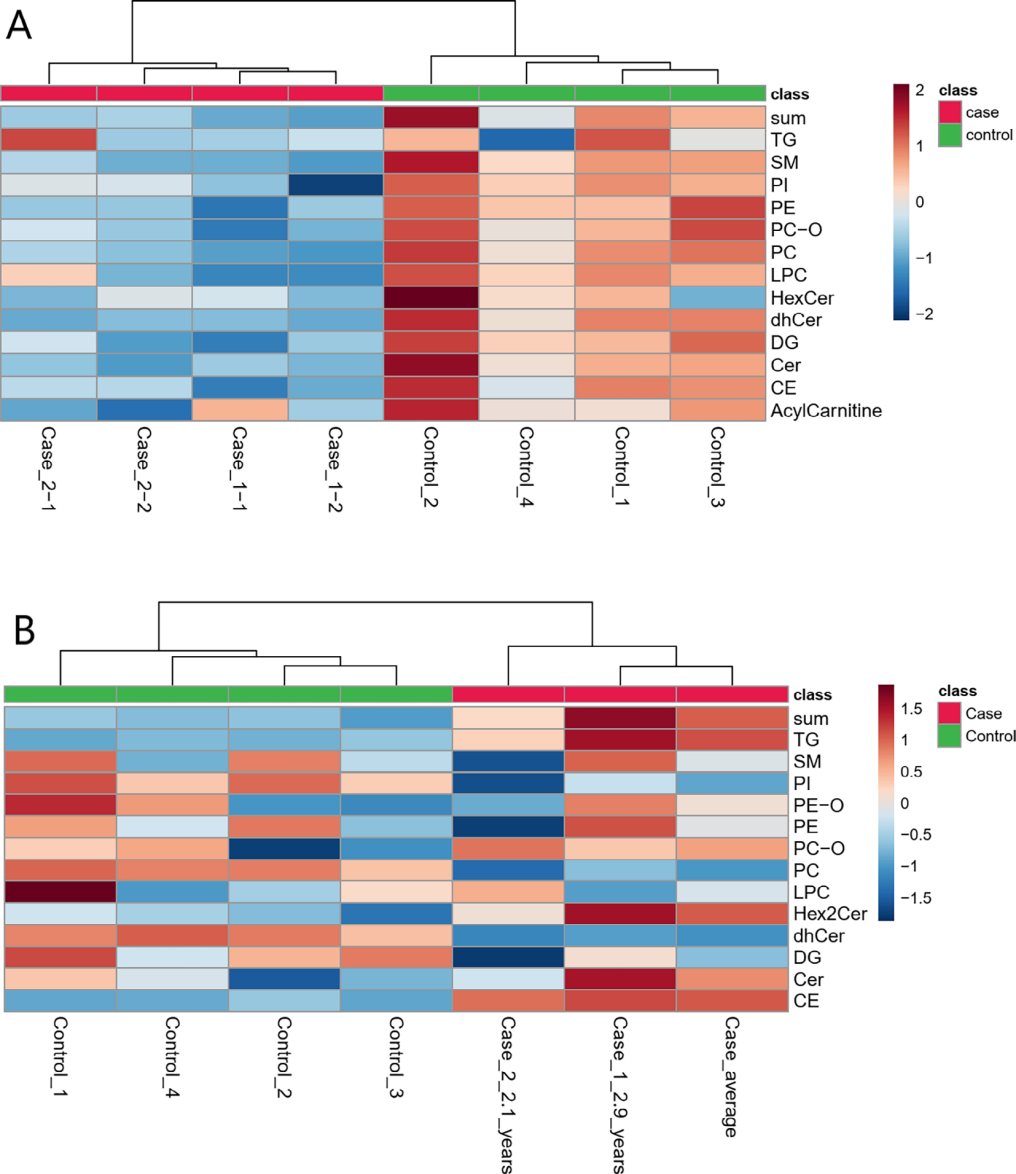
(A, B) Heatmaps showing the class‐wise differences in (A) plasma and (B) liver tissue lipid classes between cases of cholesterol ester storage disease in two field spaniels (plasma *n* = 4, two samples at different time points for both dogs; liver biopsies, *n* = 2, additionally a calculated average of the two samples was used as a third measurement to enable statistical analysis) when compared to healthy control dogs (*n* = 4, both plasma and liver tissue). (A) The amount of plasma lipids was lower for cases for all lipid classes except for triacylglycerols (TG). (B) In liver tissue cholesterol esters (CE) and TGs were consistently higher in cases compared to controls, while phosphatidylcholine, phosphatidylinositol and dihydroceramides were higher in controls. However, individual differences were detected in other liver tissue lipid classes among the two affected dogs. The sum of lipids was calculated class‐wise for each lipid class by summing the normalized peak areas for each lipid class in mass spectrometry analysis. Each column represents the results in an individual sample for each lipid class (rows). The color represents magnitude of change when compared to mean of that lipid class. For *Case 1*, the first plasma sample (Case_1–1) was obtained at the age of 3.0 years and the second plasma sample (Case_1–2) at the age of 3.7 years. For Case 2, the first plasma sample (Case_2–1) was obtained at the age of 1.7 years and the second plasma sample (Case_2–2) was obtained at the age of 2.1 years. CE, cholesteryl ester; Cer, ceramides; DG, diacylglycerol; dhCER, dihydroceramide; HexCer, hexosylceramides; Hex2Cer, dihexosylceramide; LPC, lysophosphatidylcholine; PC, phosphatidylcholine; PC‐O, ether‐linked phosphatidylcholine; PE, phosphatidylethanolamine; PE‐O, ether‐linked phosphatidylethanolamine; PI, phosphatidylinositol; SM, sphingomyelin; TG, triacylglycerols; sum, sum of all lipid classes.

Mass spectrometric detection and ultra‐high performance liquid chromatography were performed on a Sciex Exion system coupled to a Sciex 6600 QTOF (Sciex, Danaher Inc., Framingham, MA). A detailed description of lipidomic methodology is included as Data S1. The normalized peak areas of the identified lipids in plasma and liver samples were analyzed using MetaboAnalyst [1]. The lipidomic results are presented in Figures 5, 6, 7 as well as in the Data S3.

## 2 Discussion

In this case report, we describe clinical and histopathological findings as well as lipidomic changes in two Field Spaniels, in which abnormally low LAL activity was demonstrated. Cholesteryl ester storage disease (CESD) and Wolman's disease are rare genetic lysosomal storage diseases caused by lysosomal acid lipase (LAL) deficiency. LAL is an essential enzyme involved in intracellular lipid metabolism and the only enzyme known to hydrolyze cholesteryl esters and triacylglycerols in lysosomes. LAL deficiency results in intracellular accumulation of lipids, especially CEs, in various organs such as the liver, spleen, lymph nodes, and bone marrow within macrophages [2]. Deficient LAL enzyme activity is indicative of the disease in humans, and the diagnosis is most often confirmed with genetic testing [3]. In humans, a severe infantile form named Wolman's disease is due to an almost complete loss (< 1%) of LAL activity, whereas CESD occurs in conjunction with low residual enzymatic LAL activity (1%–10%) [4]. In dogs, CESD is recognized but appears to be rare, with a single case report describing histopathological findings suggestive of CESD in three related young fox terriers [5].

Clinical picture in both these cases was similar; corneal lipid accumulation was the first clinical sign noted at the age of 6 months, followed by progressive hepatosplenomegaly and weight loss in both dogs. The clinical signs mirror those of humans, where hepatomegaly and abdominal distension are common [4]. In both dogs, chronic small bowel diarrhea was also a uniform feature, possibly contributing to hypoalbuminemia and weight loss. At the tissue level, the proprial accumulation of macrophages storing lipids and cholesterol was reminiscent of a severe diffuse granulomatous enteritis, thus explaining the diarrhea and malabsorption. Signs of gastrointestinal disease are also reported in humans and are considered the result of lipid malabsorption in the small intestine and increased fecal lipid loss [3, 6].

In both dogs, mild to moderate hypocholesterolemia was detected, which was surprising, as in LAL deficiency in humans, most patients present with hypercholesterolemia. Serum cholesterol and triglyceride levels can be normal in people early in the disease, presumably as a result of malnutrition and the role of cholesterol as a negative acute phase reactant, but as the disease progresses, > 90% of people develop hypercholesterolemia [3, 7]. In our dogs, as in the cases described by von Sandersleben et al., hypocholesterolemia persisted despite advanced disease, indicating a phenotypic difference [7]. Lipidomic analysis revealed a wide array of changes in both serum and liver lipids in dogs with CESD. The main findings included significantly decreased plasma total lipids as well as a decrease in several plasma lipid classes, including CEs in affected dogs. Decrease in plasma lipids was accompanied by lipid accumulation in liver tissue, which was not fully identical in the two affected dogs. However, a consistent increase in tissue triacylglycerols and CEs was detected in both affected dogs. These findings indicate that a defective mechanism of cellular cholesterol breakdown and release occurs in affected dogs, leading to lipid accumulation intracellularly and to decreased concentrations in peripheral circulation. Furthermore, the canine hepatocytes could be more resilient to lipid and cholesterol storage, rupturing later in the disease and thus retaining a low extracellular and plasma lipid level in the animal despite intracellular storage. This is in line with the milder liver fibrosis seen in dogs in comparison to cirrhosis described in humans with CESD. The individual differences in lipid composition of liver tissue in affected dogs might be partially explained by sampling at different points of disease progression (*Case 1* 1.2 years before euthanasia and *Case 2* at the time of euthanasia). It needs to be taken into account that the affected dogs were receiving a low‐fat diet at 3/4 plasma samplings and 1/2 liver biopsy samplings, which might have influenced the lipidomics analysis results. However, as there were no marked differences between plasma samples acquired with and without a low‐fat diet in *Case 1*, the authors consider it likely that most of the changes detected in lipidomics analysis were due to the LAL deficiency. A limitation in this study was that the control group for liver tissue lipidomics was older than the affected dogs. Although the absence of liver disease was confirmed in all control dogs, the effect of aging in canine liver lipidomics is unknown and could have had an influence on the results.

In both affected dogs, normal or elevated activity of liver enzymes in serum and hypoalbuminemia were the main serum biochemistry changes, and there was no evidence of hepatic dysfunction despite advanced disease, as other functional variables, apart from albumin, remained normal (bilirubin, urea, glucose, ammonia). This is different compared to humans with LAL deficiency, where the development of hepatic fibrosis, cirrhosis, and a varying degree of hepatic dysfunction are main clinical complaints [5]. ASAT serum activity is reportedly more elevated than ALAT activity in humans, which was similar in our dogs. The relatively higher increase in ASAT, a partially mitochondrial enzyme, might be due to mitochondrial dysfunction noted in LAL deficiency [8]. Moreover, it was surprising that the liver enzyme activity in blood remained normal or only mildly increased, despite pronounced swelling of the hepatocytes noted in histopathology. This could further indicate that canine hepatocytes can tolerate marked lipid accumulation without cellular damage.

Based on the clinical and histopathological findings in the affected dogs, LAL deficiency was suspected, and enzyme activity measurements detected a severe deficiency in both dogs similar to CESD in humans [4]. In man, the degree of LAL deficiency varies, and lower enzyme activities are connected to earlier onset of clinical signs and shorter life expectancy [7]. This could agree with the findings in our dogs, since *Case 2* with lower LAL activity was euthanized at a younger age due to disease progression. It is noteworthy that the LAL activity measurement utilized had not been validated for use in dogs, and the threshold for canine CESD diagnosis was unknown. However, the authors consider the results indicative of LAL deficiency, as a marked difference in enzyme activity was detected when compared to healthy dogs. Larger future studies are necessary to identify a normal reference range for LAL activity in dogs and the diagnostic cut off for CESD.

In humans, LAL deficiency is an autosomal recessive disease caused by mutations in the LIPA gene located on the long arm of chromosome 10, and more than 40 causative mutations are described [3, 7]. The genetic background of the disease in these Field Spaniels is under investigation. However, the affected dogs in our study did not have common ancestors when evaluating the pedigree data for three previous generations. Interestingly, genome‐wide association studies and functional genomic studies have identified LIPA as a risk locus for coronary heart disease in humans, but the causal variants and mechanisms remain to be determined [9]. In affected dogs, vascular changes were not detected, contrary to humans, where aortic plaques and strokes have been reported in connection with LAL deficiency [3, 10]. Atherosclerosis, reminiscent of the vascular changes in coronary heart disease in man, can be induced in dogs through a high‐cholesterol diet and is an almost pathognomonic finding in dogs with hypothyroidism and consequent hypercholesterolemia [11]. The lack of hypercholesterolemia in the LAL‐deficient dogs could be one reason for this phenotypic difference.

Treatment of CESD in humans consists of a low‐fat diet, cholesterol‐lowering drugs, and long‐term enzyme replacement therapy using sebelipase alfa, which is recombinant human LAL [3]. In our dogs with LAL‐deficiency, a low‐fat diet was prescribed, but cholesterol‐lowering drugs were not indicated due to hypocholesterolemia. With this supportive treatment, the dogs were managed for a period of time (15 months in *Case 1* and 5 months in *Case 2*), but long‐term treatment was unsuccessful as expected. Unfortunately, sebelipase alfa is extremely expensive and therefore treatment trials using this human LAL‐enzyme replacement product were not feasible for our dogs.

## Disclosure

Authors declare no off‐label use of antimicrobials.

## Ethics Statement

Blood sampling from dogs was approved by the Board of Animal Experimentation of the Regional State Administrative Agency of Southern Finland (decision ESAVI/31506/2022). The use of client owned cadavers donated for teaching and research purposes (acquisition of control biopsies in this study) has been approved by the Viikki Campus Research Ethics Committee of the University of Helsinki, Finland (Statement 17/2021). The use of surplus biological samples for research purposes has been approved by Viikki Campus Research Ethics Committee, University of Helsinki (Statement 13/2020). Written owner consent was obtained from all owners before study participation. At the Faculty of Veterinary Medicine, University of Helsinki, the Pathology and Parasitology Unit holds all further rights to histopathology specimens submitted to analysis. This faculty policy is informed to clients in writing. Authors declare human ethics approval was not needed.

## Conflicts of Interest

The authors declare no conflicts of interest.

## Supporting information


**Data S1:** Supporting Information.

**Data S2:** Supporting Information.

**Data S3:** Supporting Information.
