# Supplementary material for: Cholesterol Ester Storage Disease in Two Field Spaniels With Lysosomal Acid Lipase Deficiency
Source: J Vet Intern Med. 2025 Aug 26;39(5):e70223. doi: 10.1111/jvim.70223 (PMC12380722; doi:10.1111/jvim.70223)
Supplement: Supplementary file 1 — Data S1: Supporting Information. [file JVIM-39-e70223-s001.pdf]

Supplementary information (S1)

Demographic data from healthy privately owned dogs included as controls in the lysosomal acid lipase measurement as well as plasma and liver tissue lipidomic analysis.

a) Lysosomal acid lipase measurement and plasma lipidomic analysis:

Dog	Breed	Sex	Age (years)	Serum biochemistry	Hematology
1	Australian shepherd	Female	5.1	Normal	Normal
2	Beauceron	Male	2.5	Normal	Normal
3	Labrador retriever	Male	3.0	Normal	Normal
4	Finnish Lapphund	Female spayed	3.2	Normal	Normal

b) Liver tissue lipidomic analysis:

Dog	Breed	Sex	Age (years)	Hematoxylin and eosin -stained histopathology slides from liver
1	Cavalier Kingcharles spaniel	Female	15.0	Absent significant liver pathology
2	American bulldog	Female	0.8	Absent significant liver pathology
3	Mixed breed	Male	9.5	Absent significant liver pathology
4	Mixed breed	Male	13.6	Absent significant liver pathology
