# Supplementary material for: Cholesterol Ester Storage Disease in Two Field Spaniels With Lysosomal Acid Lipase Deficiency
Source: J Vet Intern Med. 2025 Aug 26;39(5):e70223. doi: 10.1111/jvim.70223 (PMC12380722; doi:10.1111/jvim.70223)
Supplement: Supplementary file 2 — Data S2: Supporting Information. [file JVIM-39-e70223-s003.pdf]

Supplementary information (S2)

Lipidomics methodology

Liver homogenates were prepared by accurately weighing 10 mg of liver tissue, adding 100 µL of saline, homogenizing the sample with a tissuelyser. Plasma samples and liver homogenate were prepared using a method based on the Folch procedure, as detailed by Lamichhane et al. (2019).[1,2]

An internal standard mixture containing 2.5 µg/mL N-pentadecanoyl-D-erythro-sphingosine(d7N-tridecanoyl-D-erythro-sphinganine-d7, cholesteryl-d7 palmitate, 1-pentadecanoyl-2-oleoyl(d7)-sn-glycero-3-phosphocholine, 1-pentadecanoyl-2-oleoyl(d7)-sn-glycero-3-phosphoethanolamine, 1-oleoyl(d7)-2-hydroxy-sn-glycero-3-phosphocholine, 1-oleoyl(d7)-2-hydroxy-sn-glycero-3-phosphoethanolamine, 1-pentadecanoyl-2-oleyol(d7)-sn-glycerol, 1,3-dipentadecanoyl-2-oleyol(d7)-glycerol and N-oleoyl(d9)-D-erythro-sphingosylphosphorylcholine was prepared in chloroform:methanol (2:1, v/v).

Seven-point calibration curves with concentrations between 0.1and 5 ppm in CHCl3:MeOH (2:1, v/v) were prepared for Cholesteryl oleate , Cholesteryl linoleic acid, N-9Z-octadecenoyl-sphinganine, N-tetracosanoyl-sphinganine, N-oleoyl-D-erythro-sphingosine, 1-stearoyl-2-hydroxy-sn-glycero-3-phosphocholine, 1-oleoyl-2-hydroxy-sn-glycero-3-phosphocholine, 1-oleoyl-2-hydroxy-sn-glycero-3-phosphoethanolamine, (3-sn-phosphatidyl)choline, 1-Hexadecanoyl-2-oleoyl-sn-glycero-3-phosphocholine, 1,2-Dioctadecanoyl-sn-glycero-3-phosphocholine, 1-palmitoyl-2-oleoyl-sn-glycero-3-phosphoethanolamine, 1,2,3-Propanetriol trihexadecanoate and 2,3-Di(octadecanoyloxy)propyl octadecanoate.

The samples were prepared by spiking 10 µL of the sample with 10 µL of 0.9% NaCl and 120 µL of internal standard solution. The samples were vortexed and were left to stand on ice for 30 min. Samples were centrifuged (9400× g, 5 min, 4 °C) and 60 µL from the lower layer was diluted with 60 µL of CHCl3:MeOH (2:1, v/v).

For the liquid chromatography (LC) separation, a Shimadzu LC30 LC system (Shimadzu, Japan) equipped with an autosampler cooled to 10 °C, a column compartment heated to 50 °C and a binary pump was used. A Waters ACQUITY BEH C18 column (2.1 mm × 100 mm, 1.7 µm) was used for

chromatographic separation. The flow rate was 0.4 mL/min and the injection volume was 1 µL. The needle was washed with 10% DCM in MeOH and ACN: MeOH: IPA: H2O (1:1:1:1, v/v/v/v) + 0.1% HCOOH after each injection for 7.5 s each. The eluents were H2O + 1% NH4Ac (1M) + 0.1% HCOOH (A) and ACN: IPA (1:1, v/v) + 1% NH4Ac + 0.1% HCOOH (B). The gradient was as follows: from 0 to 2 min, 35–80% B, from 2 to 7 min, 80–100% B, and from 7 to 14 min, 100% B. Each run was followed by a 7 min re-equilibration period under initial conditions (35% B).

For data preprocessing, the raw data files were converted to a .mzml file using Bruker compass data analysis 5.1. The preprocessing was performed in MZmine. Briefly, centroid mass detection was performed, followed by ADAP chromatogram builder, chromatogram deconvolution (local minimum search), and isotopic peaks grouper with join aligner. After this, a filtering step (feature list row filter), a custom database search, an adduct search, and gap filling (peak finder) were performed. Finally, the results were exported as a CSV file. After this, lipid class-based normalization was performed using the class-based internal standards; class-based calibration curves were created. Features that were annotated and had a relative standard deviation of less than 30% in the quality control samples were selected for further processing.

References:

1. Folch J, Lees M, Sloane Stanley GH. A simple method for the isolation and purification of total lipides from animal tissues. *J Biol Chem*. 1957;226(1):497-509.
2. Lamichhane S, Kemppainen E, Trošt K. et al. Circulating metabolites in progression to islet autoimmunity and type 1 diabetes. Diabetologia. 2019; Dec;62(12):2287-2297.
