# Supplementary material for: Cholesterol Ester Storage Disease in Two Field Spaniels With Lysosomal Acid Lipase Deficiency
Source: J Vet Intern Med. 2025 Aug 26;39(5):e70223. doi: 10.1111/jvim.70223 (PMC12380722; doi:10.1111/jvim.70223)
Supplement: Supplementary file 3 — Data S3: Supporting Information. [file JVIM-39-e70223-s002.pdf]

Supplementary information (S3): Lipidomic results

Materials

Cases: Plasma (n=4) and liver tissue (n=2) samples from two Field Spaniels with cholesterol ester storage disease.

Controls: Plasma (n=4) and liver tissue (n=4) samples from healthy dogs.

Analysis: Mass spectrometry

Total amount of plasma and liver tissue lipids and cholesterol esters

The total amount of lipids and cholesterol esters (CE) was calculated as the sum of normalized peak areas (NPAs) for all identified lipids and all identified CEs and compared between cases and controls (Mann-Whitney test). The total amount of plasma lipids and plasma CEs was lower in cases (median 211.9 NPAs and median 103.2 NPAs) compared to controls (median 625.7 NPAs and median 427.7 NPAs; P=0.029 and P=0.029, respectively), while total liver tissue lipids and CEs was higher in affected dogs (median 399.1 NPAs and median 201.0 NPAs) when compared to controls (median 172.4 and median 3.0 NPAs, respectively).

Plasma and liver tissue lipids

The plasma lipids that showed the most significant differences between the cases and controls included phosphatidylcholine (PC)(18:0p/18:1), PC(36:3), PC(36:2), PC(0-34:3), and sphingomyelin (SM)(34:2). Except for triacylglycerol (TG)(53:3) and TG(49:2), all significantly altered lipids were found to be lower in the case group compared to the controls. In liver tissue, especially cholesterol esters (CE) and TGs were increased in cases when compared to controls and PC(O-32:0), CE (20:4) and CE(18:3) were most significantly increased.

Plasma and liver tissue lipid classes

The amount of plasma lipids was lower for cases for all lipid classes except for triacylglycerols (TG). In liver tissue cholesterol esters (CE) and TGs were consistently higher in cases compared to controls, while phosphatidylcholine, phosphatidylinositol and dihydroceramides were higher in controls. Individual differences were detected in other liver tissue lipid classes among the two affected dogs.

Relative distribution of lipid classes

The relative distribution of plasma lipid classes was different in cases compared to controls. While CE was the major plasma lipid class and hexosylceramides (HexCers) was the second most abundant plasma lipid class in both groups, the proportion of CEs was lower in cases compared to controls. The proportion of plasma HexCers on the other hand was larger in cases. In liver tissue, the largest lipid class in controls was PCs, while in cases the largest class was CEs, followed by sphingomyelins.

Figures 1.

Pie charts representing the relative distribution of plasma lipid classes.

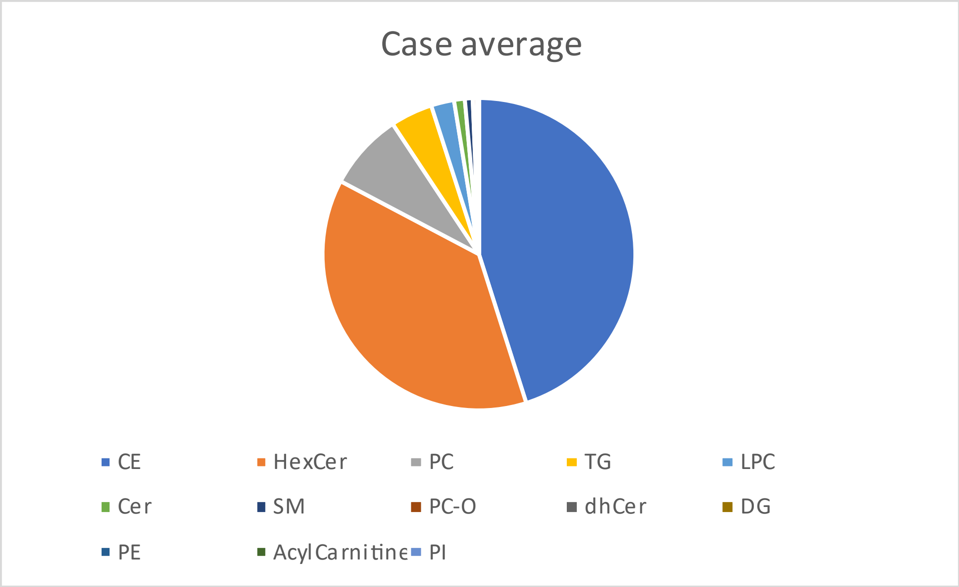

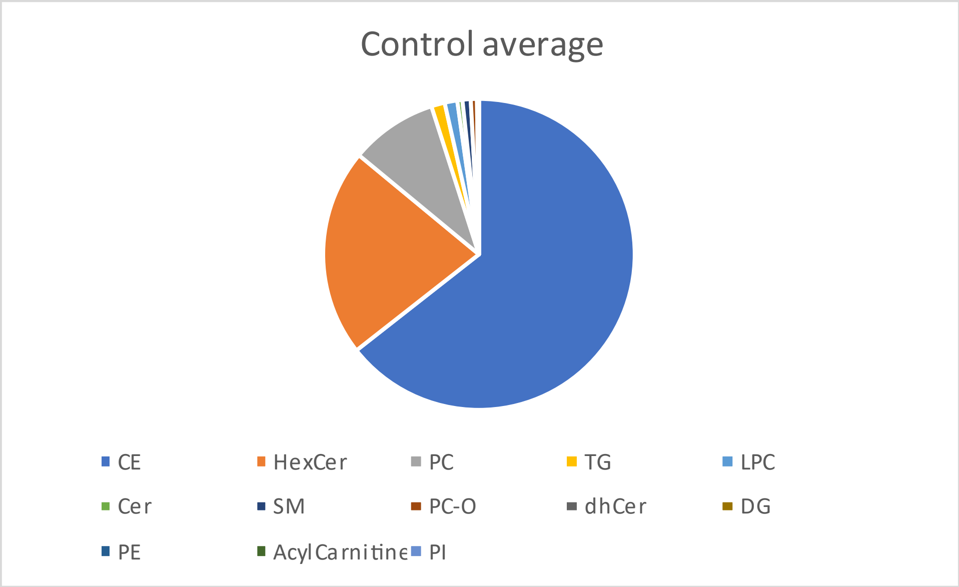

Figures 2

Pie charts representing the relative distribution of liver tissue lipid classes.

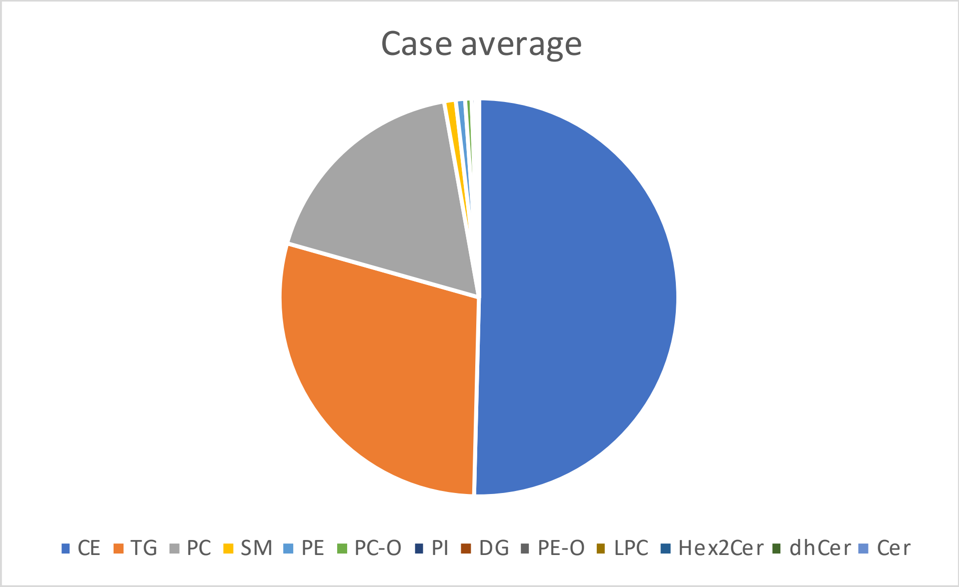

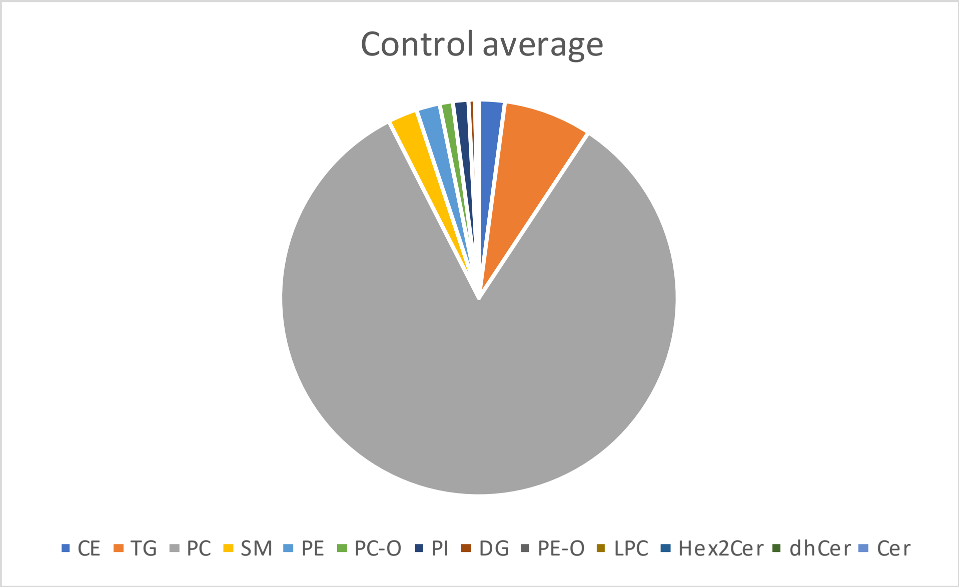

CE= Cholesteryl ester, Cer = Ceramides, DG=Diacylglycerol, dhCER=Dihydroceramide, HexCer = Hexosylceramides, Hex2Cer = Dihexosylceramide, LPC = Lysophosphatidylcholine, PC=Phosphatidylcholine, PC-O=Ether-linked Phosphatidylcholine, PE=Phosphatidylethanolamine, PE-O=Ether-linked Phosphatidylethanolamine PI=Phosphatidylinositol and SM=Sphingomyelin (SM), TG = Triacylglycerols
